# Mechanical Assessment of Denture Polymers Processing Technologies

**DOI:** 10.3390/jfb15080234

**Published:** 2024-08-21

**Authors:** Cristina Modiga, Andreea Stoia, Marius Traian Leretter, Ana Codruţa Chiş, Andreea-Violeta Ardelean, Edward-Ronald Azar, Gabriel Kapor, Daniela-Maria Pop, Mihai Romînu, Cosmin Sinescu, Meda-Lavinia Negruţiu, Emanuela-Lidia Petrescu

**Affiliations:** 1 Department of Prostheses Technology and Dental Materials, Faculty of Dental Medicine, University of Medicine and Pharmacy “Victor Babeş” Timișoara, Eftimie Murgu Sq. No. 2, 300041 Timişoara, Romania; modiga.cristina@umft.ro (C.M.); pop.daniela@umft.ro (D.-M.P.); rominu.mihai@umft.ro (M.R.); sinescu.cosmin@umft.ro (C.S.); negrutiu.meda@umft.ro (M.-L.N.); petrescu.emanuela@umft.ro (E.-L.P.); 2 Research Center in Dental Medicine Using Conventional and Alternative Technologies, University of Medicine and Pharmacy “Victor Babeş” Timișoara, Eftimie Murgu Sq. No. 2, 300041 Timişoara, Romania; andreea-violeta.ardelean@student.umft.ro (A.-V.A.); edward-ronald.azar@student.umft.ro (E.-R.A.); 3 Department of Mechanics and Strength of Materials, “Politehnica” University of Timisoara, 1 Mihai Viteazu Ave., 300222 Timişoara, Romania; andreea.stoia2@upt.ro (A.S.); gabriel.kapor@student.upt.ro (G.K.); 4 Department of Prosthodontics, Faculty of Dental Medicine, University of Medicine and Pharmacy “Victor Babeş” Timișoara, Bd. Revolutiei din 1989, Nr. 9, 300041 Timişoara, Romania; 5 Research Institute for Biosafety and Bioengineering, The King Michael I University of Life Sciences, 119 Aradului Road, 300645 Timişoara, Romania; codruta_chis@usvt.ro

**Keywords:** denture polymers, processing technologies, polymers injection, 3D printing, substractiv CAD/CAM technique, mechanical testing

## Abstract

Background: Removable prostheses have seen a fundamental change recently because of advances in polymer materials, allowing improved durability and performance. Despite these advancements, notable differences still occur amongst various polymer materials and processing technologies, requiring a thorough grasp of their mechanical, physical, and therapeutic implications. The compressive strength of dentures manufactured using various technologies will be investigated. Methods: Traditional, injection molding, and additive and subtractive CAD/CAM processing techniques, all utilizing Polymethyl methacrylate (PMMA) as the main material, were used to construct complete dentures. The specimens underwent a compressive mechanical test, which reveals the differences in compressive strength. Results: All the specimens broke under the influence of a certain force, rather than yielding through flow, as is characteristic for plastic materials. For each specimen, the maximum force (N) was recorded, as well as the breaking energy. The mean force required to break the dentures for each processing technology is as follows: 4.54 kN for traditional packing-press technique, 17.92 kN for the injection molding technique, 1.51 kN for the additive CAD/CAM dentures, and 5.9 kN for the subtractive CAD/CAM dentures. Conclusions: The best results were obtained in the case of the thermoplastic injection system and the worst results were recorded in the case of 3D printed samples. Another important aspect depicted is the standard deviation for each group, which reveal a relatively unstable property for the thermoplastic injected dentures. Good results here in terms of absolute property and stability of the property can be conferred to CAD/CAM milled group.

## 1. Introduction

Removable prostheses, as an alternative for the dental implant, have become indispensable, especially in economically deprived areas, considering the fact that the global population of older adults is projected to reach two billion by 2050 [1,2,3,4]. Given that these restorations mostly address the senior population, it is important to point out that accidental dropping is more likely to occur, leading to fractures and damages [4]. To meet this growing need, various materials have been investigated, intending to optimize the prosthesis bases while focusing on enhancing biocompatibility, resistance, and longevity of these devices [5].

The main material used for denture bases is polymethyl methacrylate (PMMA), due to its broad application and adaptability. PMMA, which is created by addition chain reaction polymerization, has several mechanical qualities that make it a good choice for prosthodontics [6]. However, the end product’s flexural strength and modulus are seriously influenced by the polymerization technique: compression molding, injecting, and additive and subtractive CAD/CAM [7].

Removable prostheses have seen a fundamental change recently because of advances in polymer materials, allowing improved durability and performance [8,9]. Despite these advancements, notable differences still occur amongst various polymer materials, requiring a thorough grasp of their mechanical, physical, and therapeutic implications [10,11,12].

The material of choice for prosthetic bases is Polymethyl Methacrylate (PMMA) because of its favorable mechanical properties and its versatility [6]. Despite its extensive application, PMMA presents certain drawbacks, including polymerization shrinkage, feeble flexural strength, and vulnerability to fractures [6]. To overcome these shortcomings, research was conducted into the use of additions and modifications to improve PMMA’s qualities, such as copolymers, plates, and fiber reinforcement [13,14,15,16,17,18,19]. Moreover, improvements in CAD/CAM fabrication processes indicate better mechanical qualities for PMMA dentures than those made using conventional techniques [20,21,22,23]. PMMA is still a good alternative for detachable prosthesis despite its drawbacks, especially when combined with cutting-edge manufacturing processes and improved materials [20,21,22,23].

A PMMA-based material was the chosen material for this paper, being the most used material by the majority of practitioners.

This research aims to compare the differences in compressive strength of complete dentures fabricated by means of four different processing technologies, using the bite record of the same patient across all technologies. The intraoral setting was optimal: healthy oral tissues with no pronounced loss of alveolar ridge tissue. The null hypothesis tested conveys that the digital processing technologies do not produce significant differences in the mechanical properties of the dentures in terms of increasing it.

## 2. Materials and Methods

Four different processing techniques, all utilizing PMMA as the main material, were used to construct complete dentures to test the null hypothesis. Eight pairs of complete dentures (sixteen complete dentures) were used as samples for each processing procedure. PMMA was chosen because of its widespread application and adaptability in the denture manufacturing process. The four selected methods for producing dentures are as follows: traditional packing-press, thermoplastic injection molding, and additive and subtractive CAD/CAM technology. The prostheses were made on didactic models, ex-vivo.

All the samples were tested in the Universal Testing Machine LBG TC100 (LBG testing equipment SRL, Azzano s.Paolo (BG) Italy). The universal testing machine is deemed to simulate compressive forces similar to those acting on the dentures in the oral cavity.

The edentulous patients are developing mostly vertical and short amplitude mastication movements. The lateral movements have minimal amplitude and could lead to an unbalanced occlusion. For these reasons, compressions forces are predominant on the complete dentures in the clinical environment [8]. In this paper, only the strength of the complete dentures on compression forces were considered.

A special Ni-Cr dental arch was created for the arch opposite that on which the denture was tested, which has contact with the artificial teeth of the denture. The denture was seated on a AlCu model, representing the supporting structures. The fit check of the dentures on the AlCu model was carried out using visual inspection of the marginal fit, as well as checking the stability of the denture on the model by assessing its resistance to movements when lateral forces are applied. Those structures allow us to test only the mechanical characteristics of the considered complete dentures.

### 2.1. Traditional Dentures

Artificial teeth are selected and fitted on the pink wax according to occlusal and functional considerations, related to used didactic models. After final adjustments, the wax setup is flasked, and the wax is eliminated through the process of boiling out. Acrylic resin is then packed into the mold and processed, resulting in the fabrication of complete dentures. The commercial product utilized was Superacryl Plus from SpofaDental (Jičín, Czech Republic). Finally, the dentures are finished, polished, and ready for testing.

**Figure 1:**
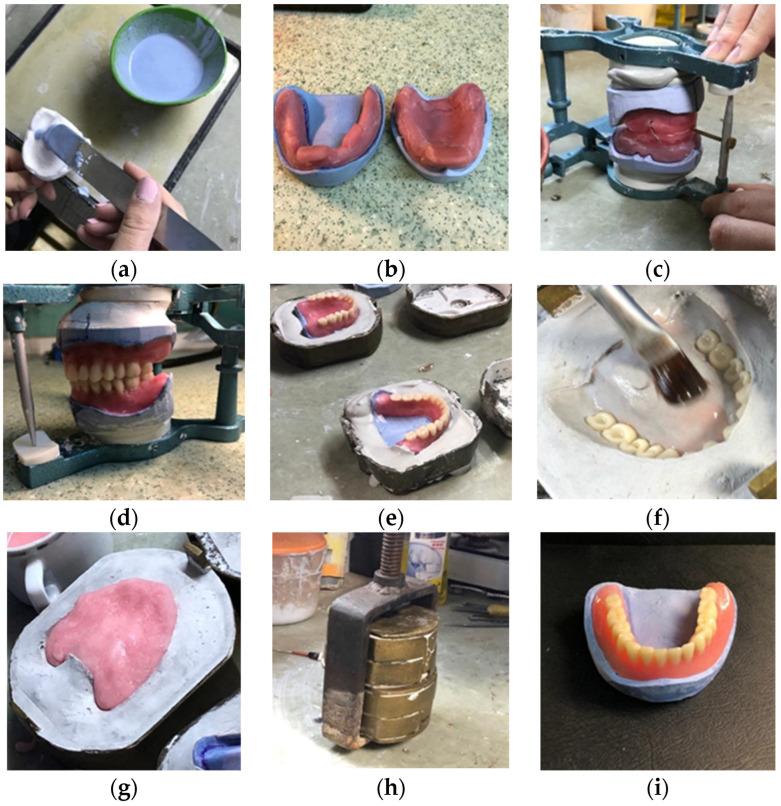
The figure illustrates the fabrication of PMMA traditional dentures: (**a**) Gypsum is poured into the preliminary impression; (**b**) Custom photopolymerizing resin impression trays are fabricated; (**c**) Wax rims have been applied to the final models; (**d**) Artificial teeth are sited; (**e**) The wax up is flasked; (**f**) Isolation prevents PMMA from sticking to the gypsum; (**g**) PMMA is packed in the void space left after the wax melted; (**h**) The denture base material filled flask is pressed to ensure proper adaptation of the material to the mold; (**i**) The flask is opened and the denture base is removed. The denture undergoes finishing and polishing processes.

In Figure 1, the working steps for obtaining a complete denture using the conventional packing-press technology are presented.

### 2.2. Thermoplastic Injected Dentures

Injectable acrylic resins have a higher density, with disadvantages pertaining to the high initial cost of the injection setup when compared to traditional dentures because they requires special equipment, such as an injector and special molds. These also have higher fracture resistance. Through industrial polymerization, the internal conversion rate is nearly 100%, resulting in a product that does not eliminate the residual monomer, which could have toxic and allergic effects in the oral cavity. The thermoplastic resin used for denture bases comes in cartridges. Injection processes are primarily employed in thermoplastic materials and rarely to thermosetting ones. The principle of plastic material injection involves pressing the molten material into the mold cavity.

**Figure 2:**
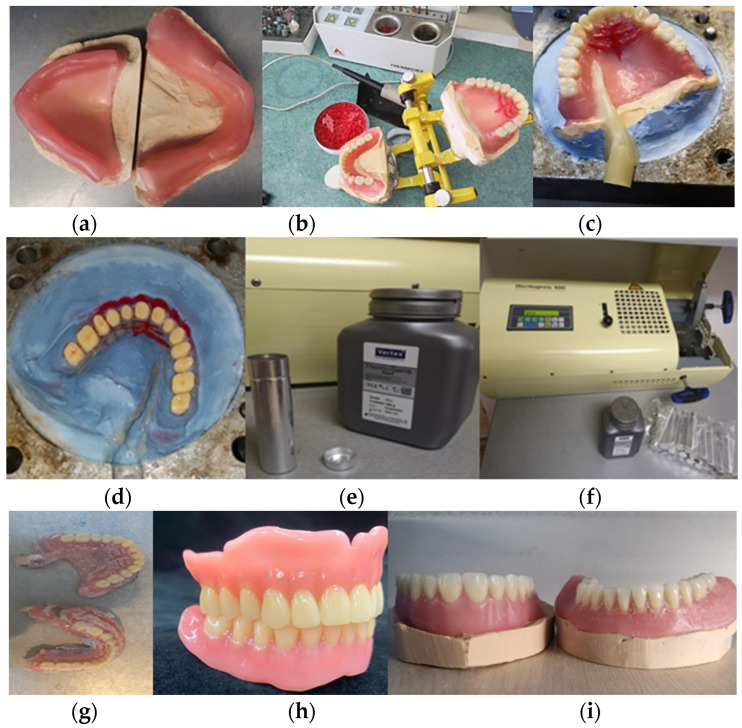
The figure illustrates the process of the fabrication of PMMA injected dentures: (**a**) Wax-up on the final cast models; (**b**) Artificial teeth are fitted onto the wax-up, after the models are mounted on a semi adjustable articulator; (**c**) The model and wax-up are flasked. Note the wax injection nozzle that will leave a void space through which PMMA will be injected; (**d**) After the wax melted a hollow space is left that will be filled by PMMA; (**e**,**f**) The injection system with the cartridges; (**g**) The dentures right after deflasking; (**h**,**i**) The final injected dentures after injection nozzle is trimmed and rough edges are smoothed out. Also, the denture undergoes finishing and polishing.

The injection molding system used in this paper was the Thermopress400 from Bredent (Senden, Germany) and the used material was Ployan IC (Bredent, Germany). Special bottles and cartridges are used for the process. The postprocessing of the dentures was performed according to the producer’s recommendations (Figure 2).

### 2.3. 3D Printed Dentures

**Figure 3:**
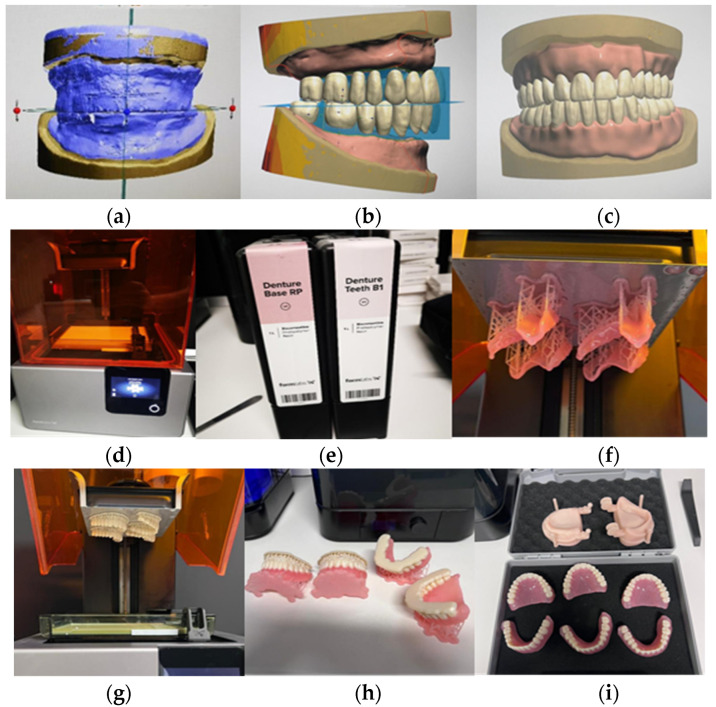
The figure illustrates the process of the fabrication of printed dentures: (**a**) Wax rims and model scan; (**b**) Teeth selection in CR (Centric Relation); (**c**) Final design of the denture base and teeth; (**d**,**e**) The printer and the resin cartridges with photopolymerizable resins; (**f**–**i**) The dentures during and right after printing.

The methodology employed for fabricating these dentures represents a departure from conventional techniques. It starts with the acquisition of a digital impression used on the same didactic models. Subsequently, the digital data are utilized by the dental technician to construct the models and fabricate the occlusal wax rims. Once the occlusal rims are created, they are subjected to scanning. Prior to this step, it is imperative for the vertical dimension of occlusion (VDO) to be established. Utilizing the tooth library within the 3Shape Dental System 2022 version 2.22.2.0, a wide array of tooth shapes and sizes are available for customization. This digital approach facilitates precise tooth selection and positioning. Illustrated in Figure 3b is the procedure of selecting denture teeth in Centric Relation (CR). After finalizing the denture base design and tooth selection, digital files are sent to the 3D printer (3D printer Form 2; FormLabs, Milwaukee, USA). These files guide the printer in creating the anatomically shaped base and ensuring accurate tooth placement in Centric Relation (CR). Cartridges containing photopolymerizable resins (Base RP for the base and B1 for teeth, FormLabs, Milwaukee, USA) are used for printing. The printer dispenses resin layers onto the platform, gradually forming highly accurate and customized dentures. This additive manufacturing technique enables intricate details to be reproduced with precision.

The postprocessing of the dentures was performed according to the producer’s recommendations.

Figure 3 provides a visual representation of the design and printing process.

### 2.4. CAD/CAM Technology

**Figure 4:**
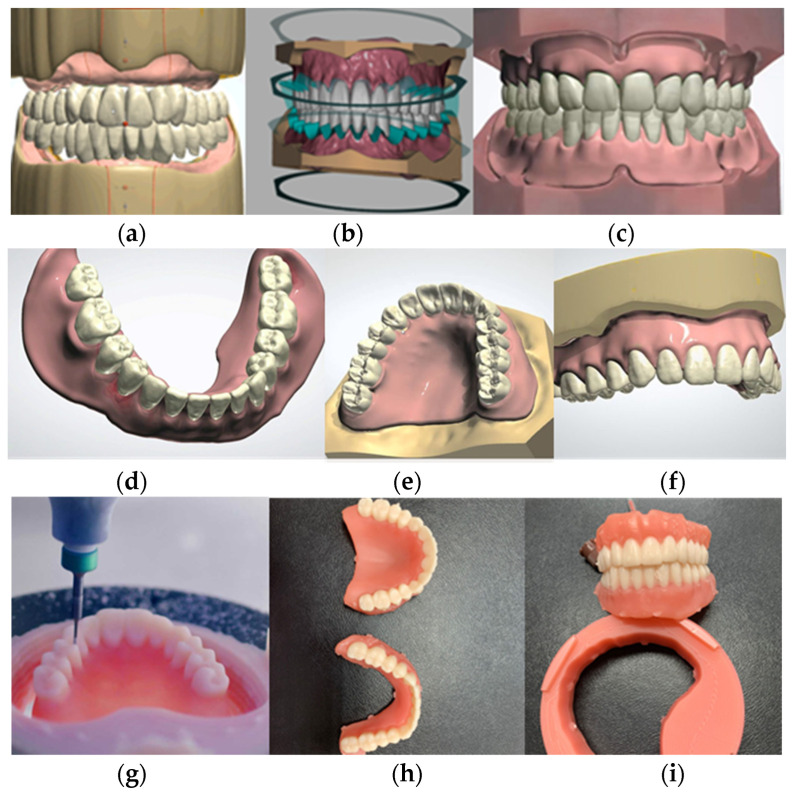
The figure illustrates the workflow for CAD/CAM dentures using Ivotion (Ivoclar): (**a**–**c**) Digital design of the dentures with teeth setting; (**d**–**f**) Individual arches can be inspected separately; (**g**) The monolithic disk is milled in PrograMill milling machine; (**h**,**i**) The milled dentures pending finishing.

The Ivotion system (Ivoclar) was employed for the subtractive CAD/CAM denture workflow. A significant innovation within this system involves the utilization of monolithic discs, wherein highly cross-linked PMMA tooth and denture base materials are merged, facilitating uninterrupted milling of both components. This integration obviates the need for laborious manual steps traditionally required for bonding teeth to the denture base, owing to the direct chemical bond formed during the polymerization process. Following the design phase in the 3Shape Dental System, the milling machine seamlessly executes the fabrication process. Subsequently, the milled dentures undergo a polishing procedure, culminating in the completion of the manufacturing process (Figure 4).

The postprocessing of the dentures was performed according to the producer’s recommendations.

### 2.5. Mechanical Testing

**Figure 5:**
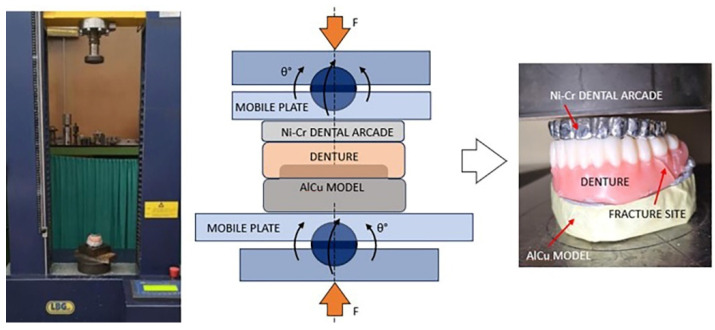
Testing setup and fractured denture after compressive mechanical test.

A total of 64 dentures were obtained and submerged in distilled water at a temperature of 37 °C for 24 h to allow for supersaturation with water, as indicated in the literature [24,25]. The thickness of the bases for all dentures was the same (2.2 mm). Subsequently, the specimens underwent mechanical compression testing. The angulated assembly consisting of the AlCu model, the denture, and the dental arch were placed between the two movable plates of a testing device in order to align the assembly with the vertical direction of the testing machine and to conform with the pure compression testing. The machine used was Universal Testing Machine LBG TC100 (LBG testing equipment srl, Azzano s.Paolo (BG) Italy), while the testing device was constructed in-house. The rotational degrees of freedom of the device (Figure 5) allows the parallelism of the machine’s plates to be maintained during testing without creating shear force that might otherwise generate lateral slipping of the assembly. The experiments were conducted to ensure that all the dentures are loaded at identical points and that they respect the same occlusion pattern. A 50 kN loading cell (0.001 kN resolution) was used to measure the compressive force while the loading velocity of the head was 2 mm/min. The tests were conducted up to the failure of the denture, the stopping criteria being a sudden dropping of force (85% of maximum). The compression test was not conducted in the densification domain of the structure.

### 2.6. Optical Microscopy

The fracture sections and the overall aspect of the denture after mechanical testing were evaluated using a stereo microscope Optika SLX-3 45x (OPTIKA S.r.l., Ponteranica (BG)-Italy) equipped with a C-B16 16 MP camera for acquiring the images. The samples were inspected as they result from the mechanical testing by means of a magnification range between 10 and 45×.

### 2.7. Statistical Analysis

The statistical analysis was performed using ‘statsmodels.stats.power’ Python library for the power analysis to determine the appropriate sample size, given a power of 0.8 (β = 0.2), α = 0.05, and an allocation ratio of 1, which indicate that a sample size of 16 dentures per group (8 mandibular and 8 maxillary) is appropriate.

Levene’s test implies significant differences in terms of variance across groups: *p* = 0.0001 for the force and *p* = 0.0000 for the energy. As such, the ‘kruskal’, ’levene’, and ‘posthoc_dunn’ methods of ‘scipy.stats’ and ‘scikit_posthocs’ Python libraries were employed for non-parametric statistical methods, as the assumption of homogeneity of variance is not met. A Kruskal–Wallis test (α = 0.001) was conducted twice to assess if there are significant differences pertaining to both the mean breaking force and energy across the four groups representing the processing technologies. Then, upon finding significant differences across the four groups, post-hoc pairwise comparisons were performed twice (for both force and energy) using Dunn’s test (α = 0.001) with a Bonferroni correction to control for multiple comparisons. Quantitative variables were expressed as Mean ± Standard Deviation and as Median (Quartile1–Quartile3).

## 3. Results

After subjecting all specimens to mechanical testing, the force-displacement curves were obtained. These were presented and interpreted instead of stress-strain curves, which cannot be computed due to the complexity of the cross-sectional area of the denture.

**Figure 6:**
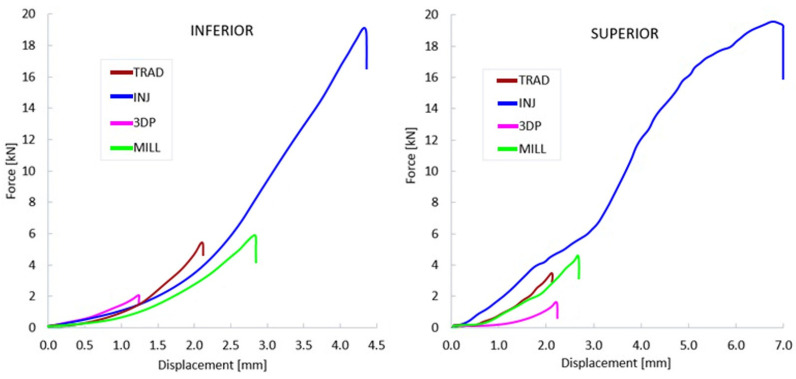
Mean Force-displacement curves for inferior and superior dentures, in compression (TRAD—traditional technology, INJ—injection technology, 3DP—3D printing, MILL—CAD/CAM milling technology).

The representative curves for each group in both the superior and inferior architectures are presented in Figure 6. Here, the load-displacement curves illustrate the elastic behavior, the absence of elastic-plastic transition, and the failure point of the construction. Significant differences can be observed between groups for both superior and inferior dentures. Regardless of the technology employed, upper dentures generally fractured at lower force values compared to lower dentures, likely due to their architectural design, which includes shape and size. The aspect of the curves reveals no clear elastic-plastic transitions for all manufacturing processes, a behavior generated by the polymeric nature of the materials.

The maximum forces at failure were extracted from the curves and additional computing of fracture energy (W, in kJ) was conducted. This parameter was computed as the area under the force-displacement curve and represents the energy absorbed by the denture during the compressive process up to failure. High values of W are associated with better performance, representing the toughness of the denture.

In the case of dentures fabricated using the traditional compression-molding technique, the force required for their fracture ranged between 2.25 kN and 7.89 kN in both architectures, with a mean value of 4.54 kN. The breaking energy exhibited values between 1.16 kJ and 6.79 kJ, with a mean of 3.58 kJ. For the thermoplastic injection technology, the values range between 14.14 kN and 28.17 kN, with a mean of 19.67 kN, for breaking force and between 17.71 kJ and 88.37 kJ, with a mean of 49.47 kJ, for breaking energy. The subtractive CAD/CAM denture, force results were seen in the range of 3.73 kN to 6.28 kN, with a mean of 5.09 kN, while the fracture energy ranges from 3.34 kJ to 6.03 kJ, with a mean of 4.63 kJ. The lowest values were observed in the case of 3D printed dentures. The fracture force required ranged between 0.72 kN and 2.23 kN, with an average of 1.51 kN, and the recorded fracture energy was between 0.28 kJ and 1.35 kJ, with a mean value of 0.81 kJ.

**Figure 7:**
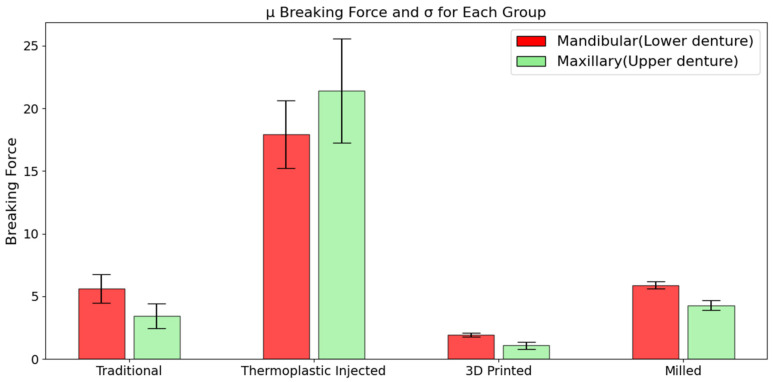
Average fracture force for each group in both architectures.

**Figure 8:**
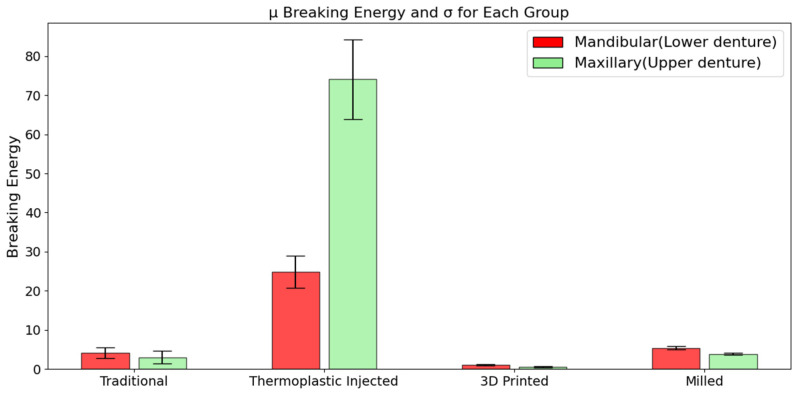
Average fracture energy for each group in both architectures.

Average fracture force and energy presented for inferior and superior dentures can be observed in the charts of Figure 7 and Figure 8; the standard deviations are also presented. Here, TRAD stand for traditional technology, INJ for injection technology, 3DP for 3D printing, and MILL for CAD/CAM milling technology. A clear domination of the INJ can be observed in both upper and lower dentures, while poor results, in terms of both force and energy, are recorded for the 3D printed dentures.

Another important aspect depicted here is the standard deviation for each group, which reveal a relatively unstable property for the thermoplastic injected dentures. Good results here in terms of absolute property and stability of the property can be conferred to CAD/CAM milled group.

The fractography of the samples reveals the external and fracture surfaces of each group. Here, particularities belonging to the manufacturing technology of the dentures can be observed.

**Figure 9:**
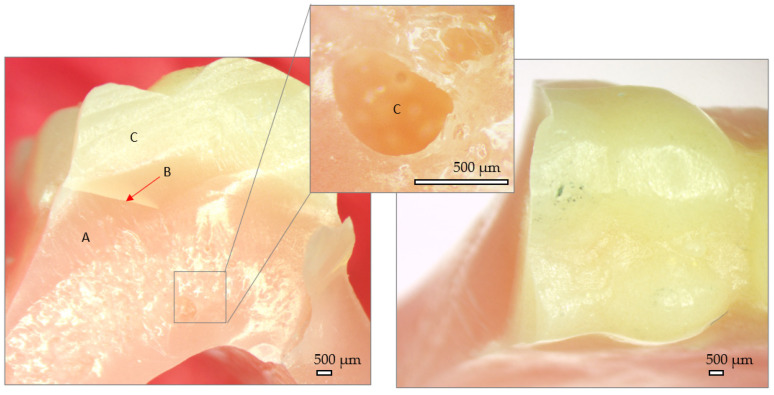
Stereo fractography of traditional denture. A—denture base, B—Interface between denture base and artificial tooth, C—artificial tooth.

In Figure 9, a cross section and external aspect of the traditional denture after mechanical testing can be observed. A very good adherence of the artificial teeth to the denture base is visible (B), with no gaps identified. In the denture base, however, reaction bubbles are visible (detail C). They lead to pore formation and are numerous in the fracture plane. The fracture has a glassy aspect with a single fracture propagation plane, which indicates a low fracture toughness property. The fracture site presents sharp edges.

**Figure 10:**
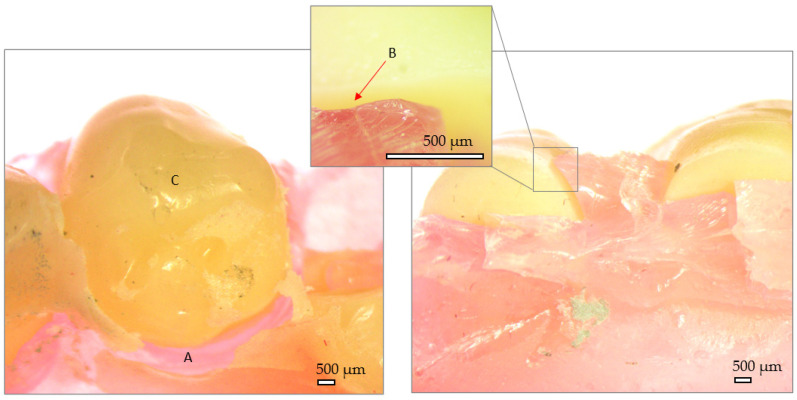
Stereo fractography of injected denture. A—denture base, B—Interface between denture base and artificial tooth, C—artificial tooth.

In Figure 10, the external aspect and fracture site of the injected denture can be observed. The denture base looks continuous, without visible pores in the fracture section. However, areas of no bonding between the artificial teeth and denture base can be observed (B). The fracture site presents no sharp edges, and a relatively high fracture toughness is manifested through small size and randomly oriented fracture planes.

**Figure 11:**
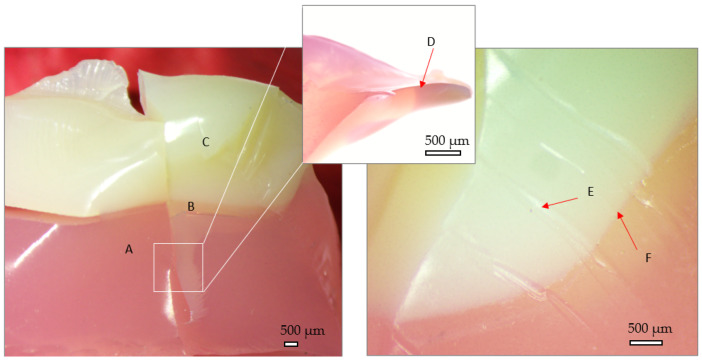
Stereo fractography of 3D printed denture. A—denture base, B—Interface between denture base and artificial tooth, C—artificial tooth; D—sharp edges; E—hackle marks formed during compression stage; F—fracture sectionThe milled denture after testing can be observed in Figure 12. Here, the milling paths in the denture base are visible (A), while visible micro scales can be observed on the artificial teeth (C). The adhesive zone presents reaction gas bubbles (B) while some areas are adhesive free. The aspect of the fracture site reveals the ductile nature of the construction.

The fractographic results of the 3D printed denture is presented in Figure 11. The sample presents a glossy aspect both at the surface and in the fracture section, which indicates a low surface roughness. There are no visible layers from the manufacturing stage, while the adhesive layer is very slim, so the transition zone is reduced. The fracture section presents hackle marks formed during compression stage (E), while the fracture surfaces present very sharp edges (D). No crushing of the artificial teeth at contact is visible.

**Figure 12:**
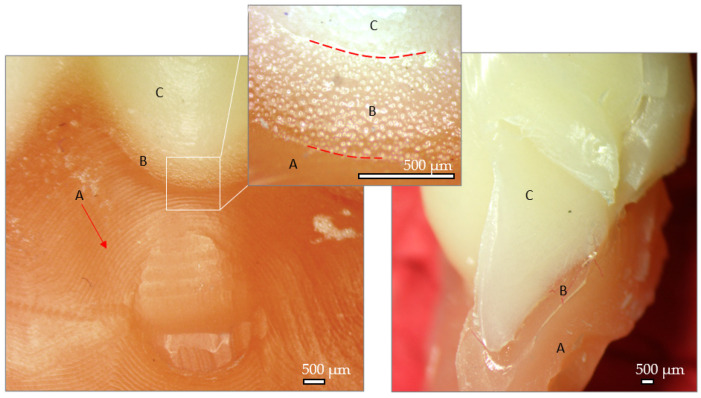
Stereo fractography of milled denture. A—denture base, B—Interface between denture base and artificial tooth, C—artificial tooth.

**Figure 13:**
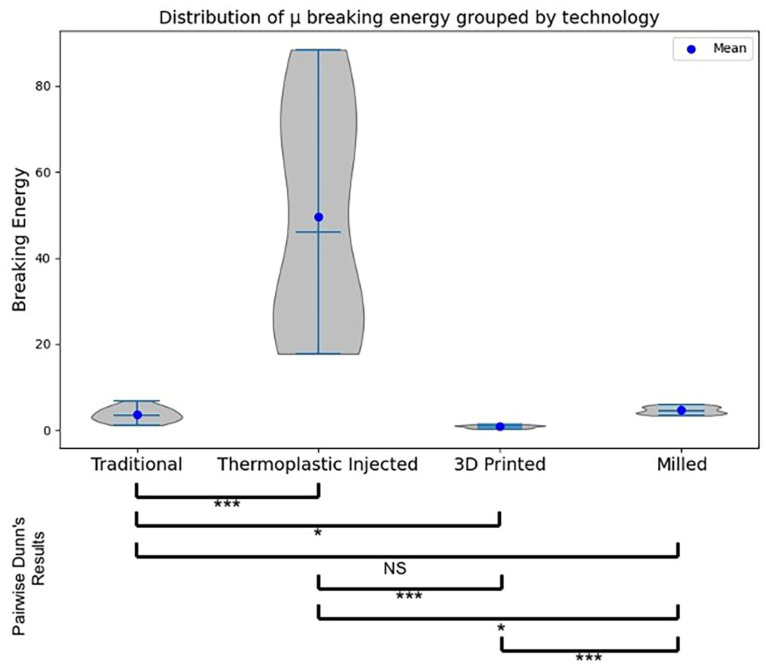
Boxplots with violin plot representations for the force (kN) values, comparative between the technological procedures. Pairwise Dunn’s test results are represented by * for *p*-value < 0.05 and *** for *p*-value < 0.001.

**Figure 14:**
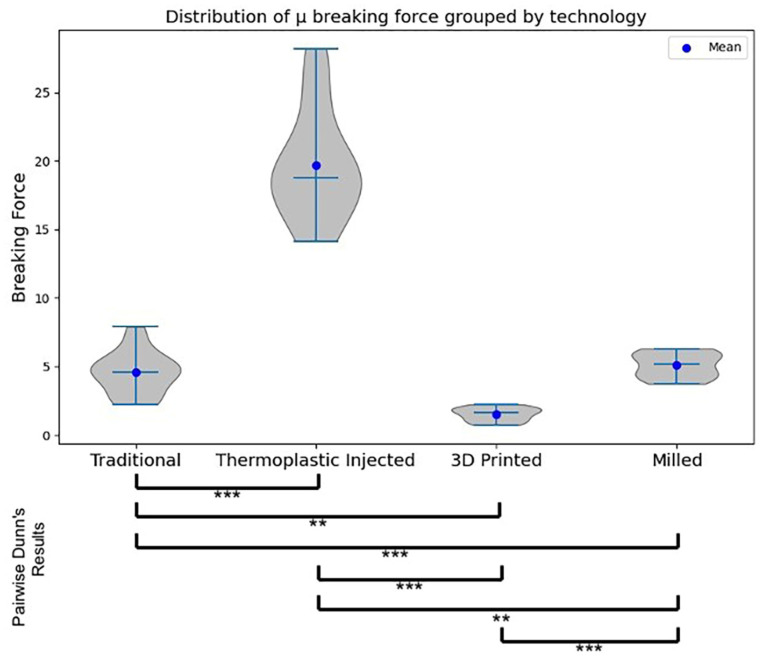
Boxplots with violin plots representing the energy (kJ) values, comparative between the technological procedures. Pairwise Dunn’s test results are represented by ** for *p*-value < 0.01, and *** for *p*-value < 0.001.

In order to ascertain notable distinctions among the samples manufactured through the four distinct polymer processing methodologies, the Kruskal–Wallis test was employed with a statistical significance threshold set at *p* < 0.001. Findings from the statistical analysis revealed that both fracture force (test H-Statistic = 53.61, *p* = 0.0000) and energy (test H-Statistic = 54.03, *p* = 0.0000) measurements exhibited a statistically significant elevation in samples generated via the thermoplastic injection procedure in comparison to all other sample groups (Table 1, Figure 13 and Figure 14).

Dunn’s test performs pairwise comparisons between all possible pairs of groups. The *p*-values obtained from Dunn’s test were adjusted using the Bonferroni adjustment to control the family-wise error rate. The Dunn post hoc test was conducted twice (one testing significant differences in breaking force and the other in breaking energy) following a significant Kruskal–Wallis test (*p* < 0.001), resulting in the adjusted *p*-value matrices.

Dunn’s test revealed the following significant differences between groups:

- Injected and 3D Printed (Force test statistic = 48.00, Force adjusted *p* = 0.0000, Energy test statistic = 47.813, Energy adjusted *p* = 0.0000)
- Injected and Traditional (Force test statistic = −26.188, Force adjusted *p* = 0.0004, Energy test statistic = −27.813, Energy adjusted *p* = 0.0001)
- 3D Printed and Milled (Force test statistic = −26.188, Force adjusted *p* = 0.0004, Energy test statistic = −27.438, Energy adjusted *p* = 0.0001)

## 4. Discussion

The null hypothesis was rejected, attesting to the fact that the technology employed in denture manufacturing does in fact have an effect on the mechanical properties of the final prosthesis.

The samples loading to failure aspect is very similar (independent of technology) in terms of loading-displacement relation and typical to the polymeric materials, which is consistent with findings from a previous study conducted by our research team [26]. Given that the denture models were identical in material, size, and shape, the observed variations in force and displacement values can be attributed to the differences in manufacturing technologies used for each group. This behavior is consistent with findings from a previous study conducted by our research group [26].

Digital dentures are increasingly becoming a possible treatment option with high expectations. Digital dentures have shown acceptable clinical performance, improved retention, reduced number of appointments, less dependence on human factors, and the ability to save patients’ records [27]. The main challenges for digital dentures include aesthetics, clinical implications, and speech difficulties [28].

Subtractive CAD/CAM dentures offer a superior treatment option compared to 3D printed dentures considering the better properties such as trueness, fitting, and strength. Having said that, its application is still limited. An understanding of these constraints and finding solutions for them are crucial before adopting digital dentures as an applicable alternative to conventional removable dentures [29]. As part of these limitations stem from the higher initial costs accrued from the technology itself, such as the digital scanner and milling machine, it is expected that the digital approach will become more accessible as these systems are better researched, leading to more manufacturers offering improved digital solutions at more competitive prices [30].

Specific studies [31] indicate that 3D-printed provisional crowns and fixed dental prostheses (FDP) made from resin materials have inferior physical properties compared to CAD/CAM milled and other conventionally fabricated provisional materials. This suggests that while 3D-printed materials may have certain advantages, they may not be as favorable in terms of their physical characteristics, such as durability, surface finish, or aesthetic qualities, when compared to their CAD/CAM milled and conventionally fabricated counterparts.

Other authors [32] compared the flexural properties of heat-polymerized (CV), CAD/CAM milled, or 3D-printed Poly (methyl methacrylate) (PMMA) [2]. Ultimate Flexural Strength (UFS), Flexural Strain (FS) (%) at Flexural Strength, and Flexural Modulus (FM) of specimens (65.0 × 10.0 × 3.3 mm) from each PMMA group (n = 6) were calculated by using the 3-point bending test. The surface roughness profiles (R) were measured before and after polishing with a contact profilometer. The Kruskal–Wallis test with post hoc analysis was performed to compare the groups (alpha = 0.05). In conclusion, the CAD/CAM milled group displayed the best flexural properties, except for Flexural Strain (FS).

Goodacre et al. [33] compared pack and press, pour, injection, and CAD/CAM milled techniques for fabricating dentures to determine which process produces the most accurate and reproducible adaptation. A definitive cast was duplicated to create 40 gypsum casts that were laser scanned before any fabrication procedures were initiated. A master denture was made using the CAD/CAM milled process and was then used to create a putty mold for the fabrication of 30 standardized wax festooned dentures, 10 for each of the conventional processing techniques (pack and press, pour, injection). Scan files from 10 casts were sent to Global Dental Science, LLC for fabrication of the CAD/CAM milled test specimens. After specimens for each of the four techniques had been fabricated, they were hydrated for 24 h and the intaglio surface laser scanned. The scan file of each denture was superimposed on the scan file of the corresponding preprocessing cast using surface matching software. Measurements were made at 60 locations, providing evaluation of fit discrepancies at the following areas: apex of the denture border, 6 mm from the denture border, crest of the ridge, palate, and posterior palatal seal. The use of median and interquartile range was used to assess accuracy and reproducibility. They found that the CAD/CAM milled fabrication process was the most accurate and reproducible denture fabrication technique when compared with pack and press, pour, and injection denture base processing techniques [33].

The purpose of another in vitro study was to compare the differences in trueness between the CAD/CAM milled and 3D-printed complete dentures [34]. Two groups of identical maxillary complete dentures were fabricated: a 3D-printed denture group (3DPD) (n = 10) and a milled denture group (MDG) (n = 10) from a reference maxillary edentulous model. The intaglio surfaces of the fabricated complete dentures were scanned at baseline using a laboratory scanner. The complete dentures were then immersed in an artificial saliva solution for a period of 21 days, followed by a second scan after immersion in saliva. A third scan (after the wet-dry cycle) was then made after 21 days, during which the complete dentures were maintained in the artificial saliva solution during the day and stored dry at night. The CAD/CAM milled complete dentures under the present manufacturing standards were superior to the rapidly prototyped complete dentures in terms of trueness of the intaglio surfaces. However, further research is needed on the biomechanical, clinical, and patient-centered outcome measures to determine the true superiority of one technique over the other with regard to fabricating complete dentures by CAD/CAM milled techniques [34].

The drawbacks of the CAD/CAM milled and 3D printed dentures were also highlighted in another paper, where material waste, high cost, need for immediate reline, and problems with VDO and phonetics were cited for CAD/CAM milled. In addition to these, 3D printed technology showed other disadvantages, such as inconsistencies with occlusion and tooth arrangement, tooth wear, need for additional visits, post insertion adjustments, overall patient dissatisfaction, and the need for remake. However, the 3D printed method is more affordable and can produce complex details with high accuracy while wasting less materials, which is considered to be one of the major benefits of this technique. In cases where retention is hindered by undesirable underlying structures, CAD/CAM milled dentures are indicated, as their bases show better overall retention, compared to the 3D printed counterparts. This is because the material comes prepolymerized under heat and pressure, so the polymerization shrinkage is minimal, which results in better fitting of the denture and thereby improving retention [30].

Saponaro found that CAD/CAM milled dentures have reduced retention, incorrect centric relation, and vertical dimension of occlusion. However, these are linked to the difficulty in obtaining a precise impression, along with lack of experience on the part of the practitioners. It was also said that more research into this was needed [35].

It was indicated that the manufacturing process would affect the mechanical properties and microbial adhesion of PMMA. CAD/CAM milled dentures showed lower surface roughness before polishing, good flexural properties, and lower microbial adhesion after 90 min of incubation when compared to 3D printed dentures. The required value of 65.0 MPa for flexural strength was exceeded. However, the surface roughness after 16 h of incubation did not vary between the groups of dentures made using different techniques. Porosity, roughness, and volumetric and linear shrinkage were cited for traditional packing press dentures. The manual skill of the operator was mentioned as the cause. CAD/CAM milled made the workflow standardized, reduced the manufacturing time, and brought the flaws that occurred due to manual skill to a minimum [30]. The CAD/CAM blocks are regularly manufactured by Hot Isostatic Pressing (HIP) from polymeric powder [36,37]. The pressure exerted in the process leads to high densification of the product, which at the structural level translates into high strength. Further CAM processing of the block into a dental structure does not lower its mechanical properties, since milling is conducted in such a way that prevents overheating of the part. The other technologies considered in the paper do not reproduce the densification level obtained by HIP, so the high mechanical properties (which are closely related to part density) cannot be reached [38].

It is imperative to acknowledge the ongoing necessity for the advancement of dental service strategies, particularly within socioeconomically disadvantaged regions. This imperative is underscored by the predominant utilization of removable prostheses among the elderly demographic. To effectively address the diverse needs within this context, it is crucial to comprehend the multifaceted influences encompassing values, attitudes, oral health literacy, and formal education that shape patterns of dental utilization [39].

Concerning the fabrication time of removable dentures, it can be stated that the 3D printing technology is the fastest, followed by the injection molding process, then the conventional packing-press procedure, with the longest fabrication time required by the CAD-CAM milled technology [40]. From the perspective of the cost price of the dentures, conventional technology is the most economical, followed by the injection molding process and then 3D printing procedures. Complete dentures obtained through CAD-CAM milled technology are the most expensive. On the other hand, regarding three-dimensional stability, the most durable dentures are those obtained through CAD-CAM milled technology, followed by those obtained through 3D printing and the thermoplastic injection process. The least dimensionally stable are those made through conventional techniques, such as manual compression molding.

There are some limitations to our research, most notably that we only looked at the mechanical analysis of compressive strength. Therefore, it would be wise for future studies to investigate other mechanical characteristics such as fatigue limit, impact strength, and surface microhardness. It should be mentioned that the experimental setup may not precisely replicate clinical conditions.

Following the experimentation and statistical analysis, the null hypothesis cannot be rejected, as statistically significant differences were observed between mechanical properties of dentures produced using distinct techniques. However, the most favorable outcomes regarding property stability were observed in dentures fabricated through CAD/CAM milling techniques. Dentures obtained via the traditional compression molding method provide satisfactory results at a significantly lower cost compared to the other discussed technologies.

## 5. Conclusions

Regardless of the technology employed, upper dentures have generally fractured at lower force values compared to lower dentures, likely due to their architectural design.

High values of fracture energy are associated with better performance (toughness) of the denture.

The clear domination of the thermoplastic injection technology can be observed in upper and lower dentures, while poor results both in terms of force and energy are recorded for the 3D printed dentures.

Another important aspect depicted is the standard deviation of each group, which reveal a relatively unstable property for the thermoplastic injected dentures. Good results in terms of absolute property and stability of the property can be conferred to CAD/CAM milled group.

The statistical analysis highlighted that the force and fracture energy recorded in the case of samples produced by thermoplastic injection procedure were significantly higher compared to all other samples.

## Figures and Tables

**Table 1:** Descriptive statistics for force and energy for the technological procedures and *p*-values resulting from the comparisons with the Kruskal–Wallis test (SD-standard deviation, Q1, Q3-the first and the third quartile).

Measured Parameter	Tehnological Procedure	Mean ± SD	Median (Q1–Q3)	* *p* Value
Force (kN)	Traditional	4.54 ± 1.52 ^a^	4.55 (3.38–5.23)	<0.001
	Thermoplastic Injected	19.67 ± 3.9 ^b^	18.79 (17.54–20.8)	
	3D printed	1.51 ± 0.48 ^c^	1.64 (1.18–1.89)	
	Milled	5.09 ± 0.88 ^a^	5.15 (4.39–5.85)	
Energy (kJ)	Traditional	3.58 ± 1.62 ^a^	3.36 (2.58–4.72)	<0.001
	Thermoplastic Injected	49.47 ± 25.77 ^b^	45.92 (26.9–68.85)	
	3D printed	0.81 ± 0.33 ^c^	0.9 (0.55–1.06)	
	Milled	4.63 ± 0.85 ^a^	4.48 (3.91–5.39)	

* Kruskal Wallis test followed by Dunn post hoc test for multiple comparison. ^a–c^ Different subscript in each row indicate a statistically significant difference between groups after post hoc analysis.

## Data Availability

The original contributions presented in the study are included in the article, further inquiries can be directed to the corresponding author.
